# Adverse drug reactions monitoring: prospects and impending challenges for pharmacovigilance

**DOI:** 10.1186/2193-1801-3-695

**Published:** 2014-11-26

**Authors:** Ram Kumar Sahu, Rajni Yadav, Pushpa Prasad, Amit Roy, Shashikant Chandrakar

**Affiliations:** Columbia Institute of Pharmacy, Tekari, Raipur, CG 493111 India

**Keywords:** Pharmacovigilance, Adverse drug reactions, Clinical trial

## Abstract

Pharmacovigilance plays a consequential role in the surveillance of adverse drug reactions, which is provoked by the drugs used to cure diseases. Adverse drug reactions (ADRs) produce detrimental or undesirable effects to the body after administration of drugs. It has been reported that the number of patients dying because of contrary effects of drugs per year increased upto 2.6-fold. Moreover, rates of hospitalization of patients are increasing owing to adverse effects of drugs. Thus, it becomes challengeable for physician, health care providers, WHO and pharmaceutical industries to resolve the associated problem of ADRs. During the clinical trial of a novel drug, it is prominent to explore the dependability of drug. In this review, we documented the details required to identify the ADRs in patients along with reported banned drugs.

## Introduction

World Health Organization (WHO) defines that adverse drug reactions (ADRs) are noxious and unwanted effects produced by the drug, when it is applied for the ailment of disease or diagnosis (Shukla et al.^2012^). The most common examples of drugs that produce ADRs include paracetamol and nimesulide (hepatotoxic effects) (Rehan et al.^2002^).

It is a well-known fact that no drug is completely free from side effects. The European commission proclaimed ADRs (Rishi et al.^2012^; Rehan et al.^2002^) till date are referred in Table 1.

**Table 1:** **ADRs report according to European Commission Impact**

Patient’s report	Percentage reported
Patients admitted in hospital	0.3% - 5%
Patient’s death reported	3.5%
Patient’s reported with ADR during their hospital stay	1.9% -6%

Before executing any new drug in the market, its clinical trial and safety database are validated for the safety profile of the drug. In various countries, whether developed or developing, the issue of ADRs is accepted to effortlessly, and thus it becomes a prime duty to develop awareness among patients about ADRs.

When a novel drug’s safety is under process, it is being constantly supervised by pharmacovigilance centres for the identification of adverse effects of the drug, if any (Beard^1992^; Mishra and Kumar^2013^). According to WHO, pharmacovigilance is a set of practices aiming at the identification, understanding and assessment of risks associate with drugs. Moreover, they take steps to control the adverse effect of drugs. Pharmacovigilance starts from the pre-marketing of new drugs and continues through the post-marketing of drugs (Lazarou et al.^1998^).

There are a bunch of examples of drugs, which have been detached as well as outlawed from the European market owing to reported adverse effects of drugs. Rosiglitazone holds the first position in the market; other well-known drugs including terfenadine, cisapride, phenylpropanolamine, rofecoxib, cerivastatin, gatifloxacin, cisapride, sibutramine and tegaserod were withdrawn because of their adverse reactions. For every drug in the market, the adverse events, if any, should be inspected in detail, and the facts should be conveyed to the people or public for elucidation of the information (Hampton^2005^; Lisa et al.^2003^; Lazarou et al.^1998^).

In contemplation to supply the news for effective drug use in population, which includes different groups of patients, such as elderly, children and diseased patients, an adequate information regarding drug’s adverse effects is required, which is achieved by a successful pharmacovigilance programmes run on that drug (Gupta and Udupa^2011^; Santosh and Tragulpiankit^2002^).

Pharmacovigilance plays several roles such as recognition, observation, assessment and documentation of drug based problems and understanding the factors producing adverse effects (Ravi Shankar et al.^2006^; Rohilla et al.^2012^). Here we tried to summarize about ADRs, and how it can be monitored by pharmacovigilance to minimize the adverse effects of drugs. Hence, this review will provide adverse events about ADRs along with the complete information of medication errors.

## Proclamations of adverse effects of drug

The USFDA data disclosed that adverse effect of drugs increased almost two times in the endmost decade. It has been noticed that significant sum of patients were pegged out because of fervent adverse effects of drugs. This report has been authenticated in the 10^th^ issue of the Archives of Internal Medicine (Livio et al.^2012^; Doheny^2009^; Aeries^1995^). The number of reports per annum proliferated upto 2.6-fold from 1998 to 2005. In 1998, the numbers of adverse events proclaimed were 34,966, which increased to 89,842 by 2005.

The number of drugs in the market producing ADRs is documented in Table 2.

**Table 2:** **List of drugs causing ADRs**

Marketed drug	Type of dosage form	Patients affected	Side effects	References
Oxycodone	Tablet- film coated and extended release	5%	Constipation, nausea, somnolence, dizziness, pruritis, vomiting, sweating, asthenia, dry mouth, headache	(Purdue^2009^)
Fentanyl	IV , patch	7.9%	Skin reactions, respiratory depression, mental changes, stroke	(Pasero^2005^; Mohammed^2012^) (Peng and Sandler^1999^, Prommer^2009^)
Clozapine	Tablets	2.7%	Cardiotoxicity, fever, chills, bodyache, flu symptoms, mouth-throat ulcers, cough, sore throat, rapid heart rate, rapid and shallow breathing.	(Cole et al.^2009^)
Methadone	Tablets	3.6%	Respiratory depression, QT prolongation, lacrimation, chilling, rhinorrhea, tachycardia, cramps, anorexia, nausea, dilated pupils, fluching	(Kung et al.^2008^) (Minino et al.^2002^)
Morphine	Injection	3%	Constipation, addiction, asphyxia, respiratory depression, renal failure, slow heartbeat, increase in B.P.	(Haupt and Jeste^2006^)
Acetaminophen	Capsules	1.5%	dark urine, loss of appetite, jaundice, damage to liver and death, difficulty in breathing, swelling of face and lips	(Huismen et al.^2002^)
Ethanercept	Syringe and subcutaneous injection	0.18%	Injection site pain, erythema reaction, rheumatoid arthritis, vestibular neuritis	(Orlando and Perkins^2002^)
Risperidone	Tablets	1.6- 1.7%	Stroke, heart failure, pneumonia, irregular heartbeat, seizure, white patches and sore in lips	(Holford^1986^)
Paclitaxel	Injectionand vials	0.01- 0.06%	Blurred vision, black stools, painful urination, ulcers, sores, red spots on skin, urticaria	(Orlando and Perkins^2002^)
Paroxetine	Tablets	0.9%	Bone pain, serious ventricular arrhythmias, agitation, hallucinations, tremors, increase in muscle tone	(Thisted et al.^1986^)
Rofecoxib	Tablets	2-3%	Increased arrhythmias, abdominal pain, tenderness, or discomfort, nausea, blood while vomiting, bloody, black, or tarry stools, unexplained weight gain, swelling or water retention, fatigue or lethargy, skin rash, itching, yellowing of skin or eyes, flu-like symptoms, or unusual bruising or bleeding.	(Krumholz et al.^2007^)
Warfarin	Tablets	1.2 -2.3%	Fatal bleeding, stroke, heart attack, abdominal pain, crawling, numbness, increase in menstrual flow, vaginal bleeding, paralysis, shortness of breath, diarrhea, skin blisters, hemorrhage, necrosis, purple toe syndrome	(Holbrook et al.^2005^)
Celecoxib	Capsules	1%	Risk of GI ulcerations, bleeding perforations, coronary artery disorder, cellulitis, angina pectoris, deep thrombo phlebritis, myocardial infarction, pneumonia, unstable angina	(Halpern^2005^)
Atorvastatin	Tablets	37 deaths	Liver damage, loss of appetite, allergic pruritis, muscles and joint pain, tendon problems, tiredness, jaundice	(Gaudreault et al.^1982^)
Misoprostol	Tablets	5-6%	Abortion, miscarriage, GI bleeding, multi organ failure, acute pain, haemodynamic instability, oesophageal necrosis, cardiac arrest, resuscitation efforts, birth defects	(Fu et al.^1998^)
Thalidomide	Capsules	10%	Somnolence, haematuria, urticaria, asthenia, pulmonary embolism, heart failure, bradycardia, tachycardia, cardiac arryhmias, deep vein thrombosis, seizures, orthostatic hypotension, birth defects (phocomelia)	(Ito et al.^2010^)
Insulin	Only in old patients injections subcutaneous insulin pump transdermal intranasal	Not available	Hypoglycemia (may result fatal if severe), low BP, irritability, fast heartbeat, convulsions, blurred visions	(FDA^2009^)
Aspirin	Tablets	25%	Excess acid secretion, stomach cramps, haemorrhage, bronchospasm, hepatitis interstitial, nephritis, inflammation of skin, allergic, abnormal liver functioning, clotted organs and tissues	(Schluter^1989^)
COX 2 inhibitors	Tablets	3- 3.9%	Myocardial infarction, fatal stroke, death from vascular events, hypertension, congestive heart failure, ulceration, bleeding from stomach, coronary artery blockage	(He et al.^2005^; Solomon et al.^2004^)
Ciprofloxacin	Tablets– extended release tablets oral suspension	0.65- 1.2%	High BP, angina, paroxysmal supraventicular tachycardia, prolonged QT interval, blood clot in brain, hepatitis, interstitial nephritis, migraine	(Carlo and Francesco^1978^)
Gentamicin	Tablets, cream, ointments, injectable	–	Severe kidney failure, nerve damage, permanent hearing loss, agitation, stomach pain, blood in urine, chest pain, stroke, coma, hallucinations, mental changes	(Carlo and Francesco^1978^)
Imipramine	Tablets	0.001% only on overdose	Slow heartbeat, abnormal heart rhythm, Low blood pressure, Inability to have an erection, hallucinations, involuntary quivering, difficult urination, nervous, confused, heart burn, diarrhea	(Delini et al.^2007^)
Fluoxetine	Capsules, tablets, liquids	0.014-0.62%	Severe blistering, peeling and red skin rash, uneven heartbeats, tremors, overactive reflexes, hallucination and seizures	(Michael and Ma^2006^)
Tetrabenazine	Tablets	0.5% only on overdose	Neuroleptic malignant syndrome, irregular heartbeats, parkinsonism, tardive dystonia, stroke sometimes, purple patches, mental changes, tightness in chest, shortness of breath, sore throat	(Jankovic and beach^1997^)
Propofol	Injectable	0.6-1.2%	Severe hypotension, bradycardia, pulmonary edema, systole, cardiac arrest, ventricular arrhythmias, respiratory acidosis, dysponea, bronchospasm	(Douketis et al.^2007^)

Scientific data indicated that different drugs were banned, and some were detached owing to the adverse effects in patients (Tables 3 and4).

**Table 3:** **Records of banned drugs**

Marketed drug	Dosage form	Banned date	Reasons	Death occurred	Ref
Terfenadine (Seldane)	Tablets	February 1998	Irregular heartbeat, stomach pain, light coloured stools, yellowing of eyes or skin, fainting, dizziness, abdominal discomfort, dry skin or itchiness, prolongation of QT interval, headache, benign prostatic hypertrophy, acute hepatitis, cholestatic hepatitis, jaundice, hepatic dysfunction	0.9-1.2%	(Ito et al.^2010^)
Mibefradil (Posicor)	Tablets	June 1998	Leg edema, hypertension, chronic angina, rhinitis, leg edema, heart stroke, headache, abdominal pain, light headedness, dyspepsia	123 death in 1 year	(Chyka et al.^2007^)
Astemizole (Hismanal)	Tablets, capsules	July 1999	Heart problems, death, cardiac arrest, QT prolongation, Torsades de pointes, ventricular arrhythmias, cardiac arrhythmias, bradycardia, hypotension	1-2%	(Minino et al.^2002^)
Cisapride (Populsid)	Tablets, oral suspension, capsules, medi-melt tablets, injections	January 2000	Fast heartbeat, convulsions, irregular heartbeat, QT prolongations, torsades de pointes, cardiac arrest, sudden death renal failure, ventricular arrhythmias	80 deaths during clinical trial	(Solomon et al.^2004^)
Rofecoxib	Tablets	November 2007	increased arrhythmias, abdominal pain, tenderness, or discomfort, nausea, blood while vomiting, bloody, black, or tarry stools, unexplained weight gain, swelling or water retention, fatigue or lethargy, skin rash, itching, yellowing of skin or eyes, flu-like symptoms, or unusual bruising or bleeding.	2-3%	(He et al.^2005^) (Solomon et al.^2004^)

**Table 4:** **Records of drugs withdrawn after ADRs observed in patient**

Marketed drug	Dosage form	Withdrawn due to	Patient’s effected	References
Gatifloxacin	Tablets, injectables	Causes hyperglycemia and liver damage	1.2%	(Carlo and Francesco^1978^)
Phenylpropanolamine	Microcapsules, tablets, sustained release tablets	Increased risk of stroke	0.01%	(Gaudreault et al.^1982^)
Propoxyphene (Darvon)	Capsule, tablet film coated	Caused fatal heart rhythm abnormalities	0.8%	(Delini et al.^2007^)
Sibutramine	Capsule	Increased cardiovascular risk	0.2%	(Schluter^1989^)
Tegaserod	Tablets	Causes increased risk of heart attack	0.6%	(Marx^2006^)
Nimesulide (below 13 years age)	Tablets, oral suspension, gel, suppositories	Caused life threatening hepatotoxic effects	1.3%	(Gaudreault et al.^1982^)
Cisapride	Tablets, oral suspension, capsules, medi-melt tablets, injections	Risk of cardiac arrhythmias	0.03%	(Solomon et al.^2004^)
Thalidomide	Capsules	Risk of teratogenicity	6-8%	(Ito et al.^2010^)
Temafloxacin	Tablets	Caused allergic reactions and haemolyticanaemia	0.002- 0.04%	(Delini et al.^2007^)
Alpidem	Tablet- film coated,	Proved to be hepatotoxic	0.6%	(Gaudreault et al.^1982^)
Tolrestat (Alredase)	Withdrawn not available	Proved as severe hepatotoxic agent	Not available	(Schonthal et al.^2003^)
Terfenadine (Seldane)	Tablet withdrawn	Caused cardiac arrhythmias	1.2%	(Orlando and Perkins^2002^)
Mibefradil (Posicor)	Tablets	Reported to cause drug interaction	123 death in 1 year	(Holford^1986^)
Tolcapone	Tablets	Hepatotoxic in nature	Not available	(Delini et al.^2007^)
Astemizole	Tablet	Interaction with other drugs	0.2%	(Holbrook et al.^2005^)
Troglitazone		Showed to be hepatotoxic	0.009%	(Solomon et al.^2004^)
Cisapride	Tablets, oral suspension, capsules, medi-melt tablets, injections	Caused cardiac arrhythmias	80 deaths during clinical trial	(Solomon et al.^2004^)
Trovafloxacin	Oral tablets	Liver failure cases reported	0.4%	(Ito et al.^2010^)
Cerivastatin	Tablets	Caused rhadomyolysis	Not available	(Chyka et al.^2007^)
Rofecoxib (Vioxx)	Tablets	Myocardial infarction were reported	2-3%	(He et al.^2005^) (Solomon et al.^2004^)
Valdecoxib (Bextra)	Tablets	Heart attack and stroke occurred	0.8%	(Halpern^2005^)
Tegaserod (Zelnorm)	Tablets	Cardiovascular ischemic events occurred followed by heart attack and stroke	0.03%	(Marx^2006^)
Aprotinin	Tablets, injection	Death occurred	Not available	(Marx^2006^)
Thioridazine	Tablets	Cardio toxicity occurred by its use	Not available	(Marx^2006^)
Sibutramine	Capsule	Cardiovascular risk increases by its use	0.2%	(Schonthal et al.^2003^)

## Documentation of ADRs

The pharmacovigilance curriculum conveyed worldwide to motivate that all suspected drug-related adverse events should be outlined. It takes interests on reports of the following:

(A) Every adverse effect suspected or occurred by new drugs and drugs of current issue.
(B) Documentation of various drugs that caused ADRs, which include death, life-threatening conditions, disability, hospitalization and congenital abnormalities.

The significant adverse reaction of any drug should be notified within seven days. The other facts related to adverse events should be informed within eight days (Bates et al.^1995^; Classen et al.^1997^). The ADR form can be collected through any pharmacovigilance centre. The filled ADR form can be submitted to the peripheral pharmacovigilance centre. After reviewing the form, the centre forwards it to the regional centre and after that it is propelled to the zonal centre (Goldman^1998^; Palaian et al.^2006^; Ravi Shankar et al.^2010^). The details are then statistically inspected and forwarded to WHO-Uppsala Monitoring Committee (UMC).

### Procedure for reporting ADRs

It is the first duty of any pharmacovigilance centre to report all suspected adverse events of the drug if found. Information regarding ADRs and the type of ADRs that should be reported are tabulated in Table 5.

**Table 5:** **Details required for reporting ADR events**

Elements in ADR reporting	Necessary information	Others	References
What should be reported	Adverse reactions of the drug, suspected drug’s details, patient’s information	Medications overdose, pharmaceutical defect, drug interactions	Goldman^1998^
Who can report	medical practitioners or health care professionals, doctors, nurses, pharmacists, assistants, pharmaceutical technicians, pharmaceutical assistants, clinical officers and other health care providers	Manufacturers, all government and private hospital’s health center	Palaian et al.^2006^
When it can be reported	Any adverse reactions if noticed should be reported as soon as possible.	**–**	Ravi Shankar et al.^2010^
How to report	Through completely filled yellow card form	**–**	Ravi Shankar et al.^2010^
Where it can be reported	Fully filled completely ADR form should be submitted to pharmacovigilance center	**–**	Palaian et al.^2006^

## Monitoring of ADRs

ADR monitoring is spelled out as the practice of continuously monitoring the undesirable effects caused using any drug. Pharmacovigilance plays an imperative impersonation in monitoring ADRs (Hall et al.^1995^; Hornbuckle et al.^1999^; Juntti and Neuvoren^2002^).

It is inherent for pharmaceutical regulators to screen their pharmaceutical products in the market and record if any suspected adverse reactions are identified. ADRs can occur by use of various pharmaceutical products, herbal drugs, cosmetics, medical devices, biological, etc. The introducing of this monitoring procedure intends at warranting that patients receive safe and beneficial medicinal products (Karch and Lasagna^1997^).

If any of the adverse events are not stated it may result in noxious and serious effects of remedial products. Thus, properly conducting ADR monitoring programmes will help to reduce the harmful effects of therapeutic products (Kessler^1993^).

### Benefits of ADR monitoring

An ADR monitoring and reporting programme can furnish following benefits:

1. It caters information about quality and safety of pharmaceutical products.
2. It initiates risk-management plans.
3. It prevents the predictable adverse effects and helps in measuring ADR incidence.
4. It instructs health care team, patients, pharmacists and nurses about adverse drug effects and creates awareness regarding ADRs.

The main objective of ADR monitoring is to disclose the quality and frequency of ADRs and to identify the risk factors that can cause the adverse reactions (Moore^2001^; Murphy and Frigo^1993^).

### ADR monitoring includes different studies for the identification of adverse events

#### Case reports

By this method, the unpredictable (bizarre) effects i.e. TYPE-B adverse drug reactions are reported.

#### Anecdotal reporting

This kind of reporting comes through reports of individual doctors when a patient suffers from the particular effect.

#### Impulsive reporting system

This method is considered as the most efficient method. Mostly, all ADR reporting programmes follow this method. Here, the effects are recorded spontaneously. With this method, both unusual and acute ADRs can be focused on and monitored (Naranjo and Busto^1981^).

#### Intensive monitoring studies

Health care members continuously watch the patients and record all the events observed when a drug or different drugs are administered. In this, defined groups of patients are screened to detect ADRs. The main disadvantage of these studies is that the population includes the minimum patients and each patient is studied for the concise period of time. Special investigations can be performed if statistical screening is incorporated in this study method (Naranjo and Busto^1981^; Nebeker and Barach^2008^).

#### Contingent studies

In these studies, patients administering similar medicines are identified, and their events are recorded. Major drawbacks of this method are that minimum patients are included and no control group is present for comparison. The contingent examinations are too expensive, and these investigations are difficult to perform on newly marketed drugs (Nissen and Wolski^2007^).

#### Case–control studies (Retrospective Studies)

In these studies, patients who have illness or disease due to the use of a drug are investigated to check if they have taken the drug. These patients are then compared with a matched control group that is similar in confounding factors but do not possess the adverse events or illness. This is a useful method in determining whether the drug has caused the adverse event or not. However, by this method, new ADRs cannot be identified (Parthasarathi et al.^2007^).

#### Case cohort studies

These studies include both prospective cohort study and retrospective case–control studies; in other words, it is the combination of both the studies (Pearson et al.^1994^).

#### Record linkage

In this method, all the records such as prescription records, patient records and hospital records are studied to identify the illness with drugs.

#### Meta analysis

It is a quantitative examination of two or more independent studies to determine the overall effect and to describe reasons in variation of study results (Prosser and Kamysz^1990^).

#### Utilitization of resident’s statistics

If a drug-induced event is very frequent and if suspicions arise for them, case–control and experimental cohort studies shall be initiated (Rao^2010^).

## Roles of pharmacovigilance in monitoring ADRs

Many incidents occurred that caused the need of laws and regulations regarding the safe use of drugs. After rofecoxib withdrawal from the European market, the FDA rules on post-market surveillance were criticized and a new system of pharmacovigilance was introduced that provided information on identified risks (Palaian et al.^2006^; Rawlins and Thompson^1981^; Yadav^2008^).

Throughout the early post-marketing period, the product might be used in different groups of people from those used in clinical trials and much larger populations might be exposed in a relatively short time. The post-marketing product is required to develop new information, which can focus on the benefits as well as risks of the product (Arnott et al.^2012^). Pharmacovigilance produces detailed information of marketed products to ensure their safe use.

The impressive pharmacovigilance planning can reduce the adverse events of drugs in patients. The most important method used in pharmacovigilance is to collect information on a drug when it is in the pre-marketing phase is by conducting a clinical trial. This study design is not optimum to determine the ADRs of the drug. Because in this approach limited numbers of patients participate and it is not necessary that the patients resemble the population in which the drug is to be used (Arora^2008^; Bahri and Tsintis^2005^), it becomes impossible to understand the mechanism and consequence of the drug in these groups. Some methods that can be helpful in the detection as well as the prevention of suspected ADRs are listed as follows.

Different study designs are included for proper pharmacovigilance study:

- Descriptive studies:
   Descriptive studies are conducted to obtain the outcome rate of drug use events in a specific population. These studies include the data of adverse events that occurred in diseased patients. Another factor included is the drug utilization study (Biswas and Biswas 2007; Biswas 2008). These studies provide data on the specific groups of patients such as children, elderly or patient with renal or hepatic dysfunction. With these data study rates of adverse events can be reported.
- Analytical studies:
   Analytical studies are performed to study related outcomes of the exposure to the drug. They can take the form of observational as well as interventional/experimental studies. There are four main types of analytical studies namely ecological, cross-sectional, cohort and case–control (Brewer and Colditz 1999).
- Observational studies:
   Observational studies provoke aspects of drug effectiveness in patients during treatment. This is in contrast with experiments, such as randomized controlled trials, where each subject is indiscriminately allocated to a treatment group or a control group (Ciorciaro et al. 1998; Jeetu and Anusha 2010; Joshi and Sapatnekar 2008).

### Methods in pharmacovigilance for monitoring of ADR

#### Passive surveillance

**Spontaneous reports** The spontaneous reporting systems were developed after the thalidomide incident. The aim of this spontaneous reporting system is to regulate and control the safety of drugs. This system is applied in the collection of post-marketing information on safety of drugs and identification of safety signals. Consequently, this system is used in the identification of signals of new, rare and serious ADRs of drugs. This system makes it easier for physicians, patients and pharmacists to report suspected ADRs to the pharmacovigilance centre (Herdiero et al.^2005^; Olsson^2008^; Rahman et al.^2007^). The pharmacovigilance centre collects all these reports and informs the stakeholders about the new reported ADRs. By this method, we can monitor all drugs in the market throughout their lifecycles (Ravindra and Vishal^2011^); (Surendra et al.^2010^).

**Case series** The case series are applied in developing a hypothesis between post-marketing drugs and its outcome (Faich^1996^).

#### Stimulated reporting

The stimulated reporting system encourages and facilitates health professionals to report ADRs in specific situations. It is very useful in generating adverse events of drugs online (Gerritsen et al.^2011^). It is effective in generating spontaneous reports of adverse events of drugs identified during the post-marketing phase. This system can assist in minimizing events by linking stimulated reporting with early post-marketing phase (Gupta^2010^).

#### Active surveillance

Active surveillance includes a pre-organized process to find out more serious adverse events, including the additional efforts to find the adverse reactions. Risk management programme is followed in this process, and more detailed information on individual adverse event reports can be obtained compared with passive surveillance (Panos et al.^2004^; Harmark and Van Grootheest^2008^; Surendra et al.^2010^; Muthiah et al.^2012^; Lobo et al.^2013^; Kshirsagar et al.^2011^).

#### Comparative observation studies

To test a hypothesis, a study has to be performed. These are the key events to evaluate the adverse events. The study can be conducted using different methods, which can be retrospective and perspective. Major types of these studies are cross-sectional studies, case–control studies and cohort studies (Bates et al.^1995^). Cross-sectional studies are conducted for ecological analysis. These are helpful in examining the prevalence of any disease at one time point. These studies are helpful to provide information between exposure of the drug and outcome in the ecological analysis.

Case–control study can easily identify the adverse events of drugs. The ADRs are determined by comparing the two distinctive groups. Case–control studies are useful when they are aimed to investigate adverse event in different groups. It is helpful in determining the absolute incidence rate of the adverse events.

Cohort studies provide data that has been collected in a routine fashion. This study can also be used to examine safety issues in specific populations such as children and patients with co-morbid conditions (Gor and Desai^2008^; Hussein et al.^1999^).

#### Earmarked clinical examinations

After pre-approved clinical trials, if sufficient risks are identified, further clinical studies are done to find or evaluate the mechanism of action for the identified adverse reactions. Pharmacokinetic and pharmacodynamic studies are applicable in determining the particular dosing, which can cause enhanced uncertainty of adverse effects in patients. Genetic testing can also be helpful in knowing which group of patients might be at an increased risk of adverse reactions. Children, elderly and patients with renal conditions might metabolize drugs in a different manner compared with patients enrolled or included in clinical trials. By these investigations events of particular interest are focused and used to determine or quantify the magnitude of the risk (Surendra et al.^2010^).

Thus, all of the above steps are linked with adverse event monitoring studies. Consequently, good safety profile of drug can be established and further suspected adverse events can be minimized and prevented by incorporating the pharmacovigilance methods for adverse drug reactions.

## Conclusions

ADRs have a perspective to provoke harmful effects in patients. Health-care workers and pharmacovigilance constrain being more conscious of perceive the ADRs in the patient. In conclusion, this study can be useful for physician to identify the ADRs in patients by applying above mentioned methods.
